# Investigation of the Interplay between Circulating Lipids and IGF-I and Relevance to Breast Cancer Risk: An Observational and Mendelian Randomization Study

**DOI:** 10.1158/1055-9965.EPI-21-0315

**Published:** 2021-09-28

**Authors:** Vanessa Y. Tan, Caroline J. Bull, Kalina M. Biernacka, Alexander Teumer, Tom G. Richardson, Eleanor Sanderson, Laura J. Corbin, Tom Dudding, Qibin Qi, Robert C. Kaplan, Jerome I. Rotter, Nele Friedrich, Uwe Völker, Julia Mayerle, Claire M. Perks, Jeff M.P. Holly, Nicholas J. Timpson

**Affiliations:** 1 Medical Research Council (MRC) Integrative Epidemiology Unit, Population Health Sciences, Bristol Medical School, University of Bristol, Bristol, United Kingdom.; 2 Population Health Sciences, Bristol Medical School, University of Bristol, Bristol, United Kingdom.; 3 IGFs & Metabolic Endocrinology Group, School of Translational Health Sciences, Learning & Research Building, Southmead Hospital, Bristol, United Kingdom.; 4 Institute for Community Medicine, University Medicine Greifswald, Greifswald, Germany.; 5 Department of Population Medicine and Lifestyle Diseases Prevention, Medical University of Bialystok, Bialystok, Poland.; 6 Novo Nordisk Research Centre, Headington, Oxford, United Kingdom.; 7 Bristol Dental School, University of Bristol, Bristol, United Kingdom.; 8 Department of Epidemiology and Population Health, Albert Einstein College of Medicine, Bronx, New York.; 9 Division of Public Health Sciences, Fred Hutchinson Cancer Research Center, Seattle, Washington.; 10 The Institute for Translational Genomics and Population Sciences, Department of Pediatrics, The Lundquist Institute for Biomedical Innovation at Harbor-UCLA Medical Center, Torrance, California.; 11 Institute of Clinical Chemistry and Laboratory Medicine, University Medicine Greifswald, Greifswald, Germany.; 12 Interfaculty Institute for Genetics and Functional Genomics, University Medicine Greifswald, Greifswald, Germany.; 13 Department of Medicine A, University Medicine Greifswald, Greifswald, Germany.; 14 Department of Medicine II, University Hospital, LMU Munich, Munich, Germany.

## Abstract

**Background:**

Circulating lipids and insulin-like growth factor 1 (IGF-I) have been reliably associated with breast cancer. Observational studies suggest an interplay between lipids and IGF-I, however, whether these relationships are causal and if pathways from these phenotypes to breast cancer overlap is unclear.

**Methods:**

Mendelian randomization (MR) was conducted to estimate the relationship between lipids or IGF-I and breast cancer risk using genetic summary statistics for lipids (low-density lipoprotein cholesterol, LDL-C; high-density lipoprotein cholesterol, HDL-C; triglycerides, TGs), IGF-I and breast cancer from GLGC/UKBB (*N* = 239,119), CHARGE/UKBB (*N* = 252,547), and Breast Cancer Association Consortium (*N* = 247,173), respectively. Cross-sectional observational and MR analyses were conducted to assess the bi-directional relationship between lipids and IGF-I in SHIP (*N* = 3,812) and UKBB (*N* = 422,389), and using genetic summary statistics from GLGC (*N* = 188,577) and CHARGE/UKBB (*N* = 469,872).

**Results:**

In multivariable MR (MVMR) analyses, the OR for breast cancer per 1-SD increase in HDL-C and TG was 1.08 [95% confidence interval (CI), 1.04–1.13] and 0.94 (95% CI, 0.89–0.98), respectively. The OR for breast cancer per 1-SD increase in IGF-I was 1.09 (95% CI, 1.04–1.15). MR analyses suggested a bi-directional TG–IGF-I relationship (TG–IGF-I *β* per 1-SD: −0.13; 95% CI, −0.23 to −0.04; and IGF-I–TG *β* per 1-SD: −0.11; 95% CI, −0.18 to −0.05). There was little evidence for a causal relationship between HDL-C and LDL-C with IGF-I. In MVMR analyses, associations of TG or IGF-I with breast cancer were robust to adjustment for IGF-I or TG, respectively.

**Conclusions:**

Our findings suggest a causal role of HDL-C, TG, and IGF-I in breast cancer. Observational and MR analyses support an interplay between IGF-I and TG; however, MVMR estimates suggest that TG and IGF-I may act independently to influence breast cancer.

**Impact:**

Our findings should be considered in the development of prevention strategies for breast cancer, where interventions are known to modify circulating lipids and IGF-I.

## Introduction

Breast cancer is a leading cause of cancer-related death (1, 2), yet approximately 23% of cases in the United Kingdom are estimated to be preventable (3). Circulating lipid and insulin-like growth factor (IGF) traits are frequently hypothesized to underlie the effect of modifiable factors such as obesity on cancer risk (4); however, the extent to which circulating lipids and IGFs interact is unclear. It is necessary to determine the potential causal relationship between lipids, IGFs and breast cancer to prioritize intervention strategies for breast cancer prevention.

Observational studies investigating the relationship between lipid profile [low-density lipoprotein cholesterol (LDL-C), high-density lipoprotein cholesterol (HDL-C), and triglycerides (TG)] and breast cancer risk have found suggestive evidence that higher HDL-C and TG is associated with lower breast cancer risk (5, 6). Mendelian randomization (MR) studies, which use genetic variants as instruments for an exposure of interest, given their randomly allocated and fixed nature (7), support a causal role for HDL-C in reducing overall breast cancer risk, whereas effect estimates for LDL-C and TG have been less consistent (8–11).

IGF-1 modulates cell growth, metabolism, and survival, and is thought to be important in cancer initiation and progression (12–14). Observationally, circulating IGF-I levels are associated with increased breast cancer risk in both pre-and postmenopausal women (13) and recent MR estimates further support causality between circulating IGF-I and breast cancer risk (15). Several randomized control trials have reported decreased circulating IGF-I levels following LDL-C lowering statin use, suggesting that perturbation of circulating lipids can alter levels of IGF-I in circulation (16–18). Intervention studies in patients with growth hormone disorders also suggest that modification of IGF-I can alter circulating lipid levels (19–21). Population-based studies examining the relationship between circulating IGF-I and lipid profile have yielded conflicting results (22–24); however, these studies may be limited by their cross-sectional design. Hence, the direction of association and whether causation exists between circulating lipids and IGF-I is still unclear.

Given evidence implicating lipids and IGF-I as potential modifiable risk factors for breast cancer (25–27), there is motivation to assess the bi-directional relationship between circulating lipids and IGF-I and to test the hypothesis that pathways from these phenotypes to breast cancer overlap. We set out to examine the causal relationships between circulating lipid traits, IGF-I and breast cancer using genetic [two-step (28) and multivariable MR (MVMR)] and cross-sectional observational study designs.

## Materials and Methods

### Study design

**Figure 1.**
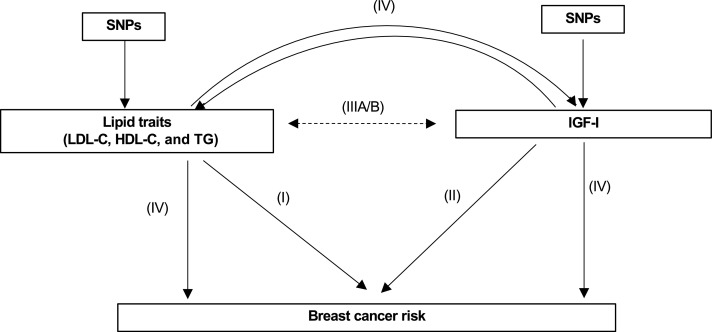
Overview of methods employed. (I) Two-sample MR analysis of the effect of lipid traits on breast cancer risk. (II) Two-sample MR analysis of the effect of IGF-I on breast cancer risk. (III) Analysis of the observational association between lipid traits and IGF-I. (IV) Multivariable MR to estimate the direct effect of IGF-I or TG on breast cancer conditioned on each other. The solid lines represent analyses using female-specific instruments, and dashed lines represent analyses using non–sex-specific instruments.

This study has four main components as outlined in Fig. 1: (I) Estimation of the causal association between lipids and breast cancer using two-sample MR. (II) Estimation of the causal association between IGF-I and breast cancer using two-sample MR. (IIIA) Analysis of the observational association between lipids and IGF-I using individual level data from the Study of Health in Pomerania (SHIP) cohort and UK Biobank (UKBB). (IIIB) Estimation of the causal association between lipids and IGF-I using a bi-directional two-sample MR. (IV) MVMR analyses to estimate the independent causal effects of lipids and IGF-I on breast cancer.

### Genome-wide association study and two-sample Mendelian randomization analyses

#### Study populations and data sources

Table 1 shows the data sources used for the two-sample MR analyses. To estimate the causal relationship between lipids or IGF-I with breast cancer using two sample MR analyses (Fig. 1, part I, II, and IV), we used summary genome wide association study (GWAS) statistics from: a female-specific GWAS of circulating lipids conducted using individual-level data from UKBB (under application #15825, *N* = 239,119; ref. 29), a female-specific meta-analysis of two IGF-I GWAS conducted by the IGF working group of the CHARGE consortium (*N* = 14,600; ref. 30) and using individual-level data from UKBB (under application #15825, *N* = 237,947; ref. 29) and a breast cancer GWAS conducted by Breast Cancer Association Consortium (BCAC, 133,384 breast cancer cases and 113,789 controls; refs. 31, 32). Analyses stratified by estrogen receptor (ER) status were also conducted (ER-positive, 69,501 cases and 105,974 controls and ER-negative, 21,468 cases and 105,974 controls). To estimate the bi-directional causal relationship between lipids and IGF-I (Fig. 1, part IIIB), we used summary GWAS statistics from: a sex-combined GWAS of circulating lipids (LDL-C, HDL-C, and TG), conducted by Global lipids Genetic Consortium (GLGC, *N* = 188,577; ref. 33), a sex-combined IGF-I GWAS meta-analysis conducted by the IGF working group of the CHARGE consortium (*N* = 30,884) and UKBB (under application #15825, *N* = 438,988). Details on the UKBB, including geographical regions, recruitment processes and other characteristics have been described previously (see Supplementary Materials and Methods for details). All individual participant data used in this study were obtained from the UKBB study, who have obtained ethics approval from the Research Ethics Committee (REC; approval no.: 11/NW/0382) and informed consent from all participants enrolled in UKBB.

**Table 1.** Data sources used for two-sample Mendelian randomization (MR) analyses.

Exposure/outcome	Consortium or cohort study	Participants (*N*)	Part I: Two-sample MR analysis of the causal effect of lipids on breast cancer	Part II: Two-sample MR analysis of the causal effect of IGFs on breast cancer	Part IIIB: The bi-directional causal association between lipids and IGFs	Part IV: Multivariable MR analyses to estimate the direct effect of IGF-I or TG on breast cancer
IGF-I	IGF working group of the CHARGE consortium	30,884 (sex-combined GWAS)^a^			X	
		14,600 (female-specific GWAS)^b^		X		X
	UKBB	438,988 (sex-combined GWAS)^a^			X	
		237,947 (female-specific GWAS)^b^		X		X
Lipid traits (LDL-C, HDL-C, and TG)	GLGC	188,577 (sex-combined GWAS)			X	
	UKBB	LDL-C:238,861; HDL-C:217,373; TG:239,119 (female-specific GWAS)	X			X
Breast cancer	BCAC	Overall: 133,384 cases and 113,789 controls	X	X		X	
		ER-positive: 69,501 cases and 105,974 controls	X	X			
		ER-negative: 21,468 cases and 105,974 controls	X	X			

Abbreviation: *N*, sample size.

^a^Sex-combined IGF-I GWAS conducted by IGF working group of the CHARGE consortium was combined with those from the sex-combined IGF-I GWAS conducted using individual-level data from UKBB.

^b^Female-specific IGF-I GWAS conducted by IGF working group of the CHARGE consortium was combined with those from the female-specific IGF-I GWAS conducted using individual-level data from UKBB.

### Lipids GWAS in UKBB

LDL-C, HDL-C, and TG were measured using enzymatic selective protection, enzyme immunoinhibition, and GPO/POD methods, respectively. The lipid measures were standardized using inverse rank normalization such that the mean was 0 and standard deviation was 1. Given that the summary estimates from BCAC described breast cancer in females only, we conducted female-specific GWAS of LDL-C (*N* = 238,861), HDL-C (*N* = 217,373), and TG (*N* = 239,119) in UKBB female participants of European descent on K-means clustering of genetic ancestry data (*K* = 4) after standard exclusions including withdrawn consent, mismatch between genetic and reported sex and putative sex chromosome aneuploidy (35, 36). We identified SNPs associated with LDL-C, HDL-C, and TG using the BOLT-LMM (linear mixed model) software (37). Analyses were adjusted for age and a binary variable denoting the genotyping chip individuals were allocated to in UKBB (the UKBB Axiom array or the UK BiLEVE array).

### IGF-I GWAS meta-analysis

IGF-I was measured in 468,384 individuals in UKBB using the chemiluminescent immunoassay (DiaSorin Ltd.). IGF-I measures were standardized using inverse rank normalization, such that the mean was 0 and SD was 1. We conducted a sex-combined GWAS for IGF-I (*N* = 438,988) in UKBB participants of European descent using the same GWAS pipeline as the lipid GWAS as described above. Analyses were adjusted for age, sex, and a binary variable denoting the genotyping chip individuals were allocated to in UKBB (the UKBB Axiom array or the UK BiLEVE array). SNP effect estimates and their SEs from the IGF GWAS in UKBB were combined with those from the IGF-I GWAS (*N* = 30,884) conducted by IGF working group of the CHARGE consortium (30) by inverse-weighted meta-analysis using GWAMA (38). Given that the summary estimates from BCAC and UKBB described breast cancer in females only, we also conducted a female-specific GWAS of IGF-I (*N* = 237,947) in UKBB using BOLT-LMM. Analyses were adjusted for age and a binary variable denoting the genotyping chip individuals were allocated to in UKBB (the UKBB Axiom array or the UK BiLEVE array). SNP effect estimates and their SEs were then combined with those from the female-specific IGF-I GWAS conducted by the IGF working group of the CHARGE consortium (*N* = 14,600; ref. 30).

#### Selection of genetic instruments for MR analyses

##### Lipid instruments:

To investigate the causal association between lipids and breast cancer risk (Fig. 1, part I), we identified 135, 214, and 203 independent SNPs [clumped on the basis of a linkage disequilibrium (LD) *r*^2^ < 0.001 and 1Mb window] associated with LDL-C, HDL-C, and TG, respectively, at *P* < 5e^−08^ from the female-specific lipid GWAS conducted in UKBB (described above). Because of the complex overlapping nature of the lipid traits, genetic variants are commonly associated with more than one lipid trait. To disentangle the roles of LDL-C, HDL-C, and TG, we also used MVMR which was developed to estimate the direct effect of various correlated risk factors when conditioned on one another in a single model (see Supplementary Materials and Methods for more details; ref. 39). For the MVMR methods, we included all female-specific GWAS-associated SNPs for LDL-C, HDL-C, and TG in the model (Supplementary Table S1).

For the bi-directional MR analyses investigating the causal relationship between lipids and IGF-I (Fig. 1, part IIIB), we selected 76, 86, and 51 independent SNPs associated with LDL-C, HDL-C, and TG at *P* < 5 × 10^−8^ from the sex-combined lipid GWAS by GLGC (*N* = 188,577; ref. 33). As effect estimates taken from overlapping datasets can be biased in the direction of the null for two-sample MR analyses, we chose to use results from the sex-combined lipid GWAS conducted by GLGC and not UKBB for this analysis as the IGF-I instruments were derived from a IGF-I GWAS meta-analysis, which included data from UKBB (female-specific lipid GWAS was not available from GLGC and thus not used for this analysis). For the MVMR methods, we selected 185 SNPs (*r*^2^ < 0.2) associated with LDL-C, HDL-C, and TG (*P* < 5 × 10^−8^) from the lipid GWAS by GLGC (*N* = 188,577; ref. 33; Supplementary Table S1).

##### IGF-I instruments:

For the MR analyses investigating the causal association between IGF-I and breast cancer risk (Fig. 1, part II), we identified 278 independent (clumped on the basis of a *r*^2^ < 0.001 within a 1Mb window) female-specific SNPs associated with the IGF-I at *P* < 5 × 10^−08^ from the female-specific IGF-I GWAS meta-analysis (Supplementary Table S2; described above).

To assess the causality and direction of association between lipids and IGF-I (Fig. 1, part IIIB), we identified 476 independent SNPs associated with IGF-I at the conventional GWAS threshold (*P* < 5 × 10^−08^), within 1MB and at *r*^2^ < 0.001 from the sex-combined IGF-I GWAS meta-analysis (Supplementary Table S2; described above). Sex-combined estimates for IGF-I were used as sex-specific lipid GWAS was not conducted by GLGC.

#### Statistical analyses

We examined the association of lipids and IGF-I with overall, ER positive and ER negative breast cancer using SNP estimates from the female-specific GWAS of lipids, IGF-I, and breast cancer (Fig. 1, parts I and II). Details of the SNPs included in each analysis, and proxies used, are provided in Supplementary Tables S1 and S2. Summary statistics were harmonized using the harmonize_data function within the TwoSample MR R package (40). All GWAS were assumed to be coded on the forward strand and harmonization was confirmed as consistent using option 2 of the “action” argument. Univariable causal estimates were combined using the inverse-variance weighted (IVW) method (41). We performed the following sensitivity analyses, each robust to some form of potential unbalanced horizontal pleiotropy: (i) MR-Egger regression method (42) to test overall directional pleiotropy and provide a valid causal estimate, taking into account the presence of pleiotropy and; (ii) weighted median method (43), which provides a consistent estimate of causal effect if at least 50% of the information in the analysis comes from variants that are valid instrumental variables. Because of the complex overlapping nature of the lipid traits, we also performed multivariable IVW MR and MVMR Egger analyses to disentangle the roles of LDL-C, HDL-C, and TG in breast cancer. As each of these sensitivity analyses make differing pleiotropy assumptions, consistency of causal effect estimates was interpreted to strengthen conclusions.

We examined the bi-directional relationship between lipid and IGF-I (Fig. 1, part IIIB) using SNP estimates from the sex-combined GWAS of lipids conducted by GLGC and IGF-I GWAS meta-analysis (described above) and the MR models described above. We performed the MR Steiger directionality test (44) to determine whether the observed observations were directionally causal based on the variance explained by the genetic instruments in the exposure and outcome and tests if the variance in the outcome is less than the exposure. We also performed LD score regression to look at the genetic correlation between lipids and IGF-I (45).

MVMR was conducted as an extension of the IVW method to test the hypothesis that circulating IGF-I may act as an intermediate factor in any reported association between circulating lipids and breast cancer, or vice versa (Fig. 1, part IV). For the MVMR analyses, we fitted a model with LDL-C, HDL-C, TG, and IGF-I. Two-sample conditional *F*-statistics were estimated to provide some assessment of instrument strength of each exposure when accounting for the prediction of other exposures in the multivariable model using the MVMR R package by Sanderson and colleagues (http://github.com/WSpiller/MVMR; refs. 46, 47).

In each instance, MR estimates are interpreted as the change in outcome per SD unit change in the exposure. Estimates for breast cancer outcomes reflect ORs. All MR analyses were performed using the MR-Base “TwoSampleMR” package (40). All other statistical analyses were performed using Stata version 14 (StataCorp) or R version 3.2.4.

### Observational analyses

#### Study populations

##### SHIP participants:

Observational analyses (Fig. 1, part IIIA) of the relationship between lipids and IGF-I were examined in a cross-sectional study within SHIP (48), a population-based project conducted in Northeast Germany (see Supplemental Materials and Methods for details). All participants underwent standardized medical examination, blood sampling, and extensive computer-aided personal interview. Data on sociodemographic characteristics and medical histories were collected. This study includes unrelated individuals with both lipid and IGF-I measurements (*N* = 3812; these data are described in detail in the Supplementary Materials and Methods). All participants gave written informed consent and the study conformed to the principles of the Declaration of Helsinki as reflected by an a priori approval of the Ethics Committee of the University of Greifswald.

##### UKBB participants:

Replication analyses to investigate the observational relationship between lipids and IGF-I were examined in a cross-sectional study within UKBB (under application #16009; described above and in the Supplementary Materials and Methods; ref. 29). We included individuals with both lipid and IGF-I measurements in this study.

#### Statistical analysis

Observational associations between lipids and IGF-I were assessed in the SHIP and UKBB using linear regression. Fully adjusted models included age, sex, smoking status, body mass index (BMI), and diabetes status. Associations of lipids and IGFs with potential confounders were estimated using linear regression.

## Results

### Part I: Two-sample MR analysis to estimate the causal effect of lipids on breast cancer

**Figure 2.**
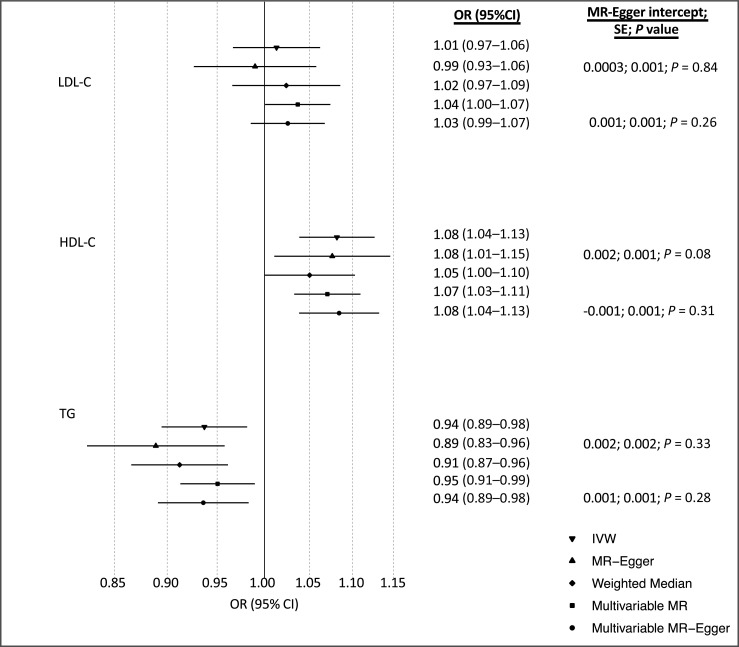
Estimates of the causal relationship between circulating lipid traits and overall breast cancer in BCAC and UKBB. The forest plot shows the estimate of the causal effect of LDL-C, HDL-C, and TG on overall breast cancer risk using summary data from the BCAC (*n* = 133,384 cases and 113,789 controls). Horizontal lines represent the 95% CIs.

The univariable IVW analyses found little evidence that LDL-C was associated with breast cancer [OR = 1.01; 95% confidence interval (CI), 0.97–1.06; *P* = 0.59]. There was evidence that HDL-C was associated with increased odds of overall breast cancer (OR = 1.08; 95% CI, 1.04–1.13; *P* = 0.0002) and TG was associated with decreased odds of overall breast cancer (OR = 0.94; 95% CI, 0.89–0.98; *P* = 0.01; Fig. 2). Estimates of all causal associations between lipids and overall breast cancer are shown in Fig. 2. Sensitivity analyses using methods that take into account potential genetic pleiotropy did not result in substantive changes in the estimates.

When assessed together using MVMR, the estimated causal odds ratio from multivariable IVW for LDL-C, HDL-C, and TG were 1.04 (95% CI, 1.00–1.07; *P* = 0.05), 1.07 (95% CI, 1.03–1.11; *P* = 0.0002), and 0.95 (95% CI, 0.91–0.99; *P* = 0.01), respectively (Fig. 2). The estimated causal odds ratios from the MVMR-Egger analyses was similar to the multivariable IVW analyses for LDL-C, HDL-C, and TG, with little evidence of directional pleiotropy (LDL-C intercept = 0.001; SE = 0.001; *P* = 0.26; HDL-C intercept = −0.001; SE = 0.001; *P* = 0.31; TG intercept = 0.001, SE = 0.001; *P* = 0.28).

We also investigated the relationship between circulating lipids and breast cancer, stratified by ER status (Supplementary Fig. S1). There was little evidence that LDL-C was associated with associated with ER-positive or ER-negative breast cancer from univariable IVW and multivariable IVW analyses. For HDL-C, the estimated causal ORs from univariable IVW (OR = 1.07; 95% CI, 1.01–1.12; *P* = 0.01) and multivariable IVW analyses (OR = 1.06; 95% CI, 1.01–1.10; *P* = 0.01) had similar direction and magnitude of association, with both analyses suggesting that HDL-C increases odds of ER-positive breast cancer. There was also evidence from both univariable IVW (OR = 1.10; 95% CI, 1.04–1.17; *P* = 0.002) and multivariable IVW analyses (OR = 1.08; 95% CI, 1.03–1.14; *P* = 0.004) that HDL-C increases odds of ER-negative breast cancer risk. For TG, the estimated causal odds ratios from univariable IVW (OR = 0.94; 95% CI, 0.89–0.99; *P* = 0.03) and multivariable IVW analyses (OR = 0.94; 95% CI, 0.90–0.99; *P* = 0.01) were consistent, with both analyses suggesting that TG decreases odds of ER-positive breast cancer. However, for TG, there was evidence of directional pleiotropy from MVMR-Egger analyses (intercept = 0.002; SE = 0.001; *P* = 0.01). Sensitivity analyses using univariable MR-Egger, weighted median and MVMR-Egger did not result in substantive changes in the estimates.

### Part II: Two-sample MR analysis to estimate the causal effect of IGF-I on breast cancer

IGF-I was associated with increased odds of overall breast cancer (IVW OR = 1.09; 95% CI, 1.04–1.15; *P* = 0.001) from IVW analyses. Estimates from MR-Egger and weighted median analyses were consistent with the IVW estimates (Table 2).

**Table 2.** Estimates of the causal relationship between IGF-I and breast cancer.

				Main analysis		Sensitivity analyses				
				IVW		Weighted median		MR-Egger		
Exposure	Consortium	Outcome	*N*	OR^a^ (95% CI)	*P*	OR^a^ (95% CI)	*P*	OR^a^ (95% CI)	*P*	MR-Egger intercept (SE; *P* value)
IGF-I	BCAC	Overall breast cancer	247,173	1.09 (1.04–1.15)	0.001	1.08 (1.02–1.14)	0.01	1.09 (0.97–1.21)	0.14	0.0002 (0.002; *P* = 0.91)
	BCAC	ER-positive breast cancer	69,501	1.09 (1.02–1.15)	0.01	1.07 (0.99–1.15)	0.07	1.10 (0.97–1.25)	0.12	−0.001 (0.002; *P* = 0.77)
	BCAC	ER-negative breast cancer	21,468	1.04 (0.96–1.12)	0.37	1.04 (0.93–1.17)	0.46	1.13 (0.96–1.33)	0.16	−0.003 (0.003; *P* = 0.26)

Abbreviation: *N*, sample size.

^a^Associations are per 1 SD unit increase in IGF-I.

Using data from BCAC, we investigated the relationship between IGF-I and breast cancer by ER-status. The odds of ER-positive breast cancer and ER-negative breast cancer was 1.09 (95% CI, 1.02–1.15; *P* = 0.01) and 1.04 (95% CI, 0.96–1.12; *P* = 0.37), respectively, from IVW analyses (Table 2).

### Part IIIA: The observational association between lipids and IGFs

Study characteristics of the SHIP and UKBB study are shown in Supplementary Table S3. In SHIP, the mean (SD) LDL-C, HDL-C, and TG levels were 3.57 (1.16) mmol/l, 1.45 (0.44) mmol/l, and 1.82 (1.30) mmol/l, respectively. The mean (SD) IGF-I was 142.1 (57.6) ng/mL.

The observational associations between circulating lipids and IGF-I using data from SHIP are shown in Supplementary Table S4. In the unadjusted analyses, a SD unit increase in LDL-C, HDL-C, and TG was associated with a −0.11 (95% CI, −0.14 to −0.08; *P* = 2.27 × 10^−12^), 0.02 (95% CI, −0.01 to 0.05; *P* = 0.29), −0.16 (95% CI, −0.19 to −0.13; *P* = 2.26 × 10^−22^) SD unit change in IGF-I levels, respectively. Circulating lipids and IGF-I were associated with potential confounders of a lipid- or IGF–breast cancer relationship, including age, sex, smoking status, diabetes status, and body mass index (Supplementary Table S5). In the fully adjusted model, a SD unit increase in LDL-C, HDL-C, and TG was associated with a 0.03 (95% CI, 0.004–0.06; *P* = 0.03), −0.05 (95% CI, −0.08 to −0.02; *P* = 0.001) and −0.06 (95% CI, −0.09 to −0.04; *P* = 1.5 × 10^−5^) SD unit change in IGF-I levels, respectively (Supplementary Table S4).

We undertook observational analyses using data from UKBB (Supplementary Table S4). In unadjusted analyses in UKBB, a SD unit increase in LDL-C, HDL-C, and TG was associated with a −0.01 (95% CI, −0.014 to −0.006; *P* = 2.08 × 10^−19^), −0.03 (95% CI, −0.032 to −0.028; *P* = 8.9 × 10^−102^), and −0.05 (95% CI, −0.054 to −0.046; *P* = 9.37 × 10^−204^) SD unit change in IGF-I levels, respectively. The association between LDL-C and TG with IGF-I is directionally consistent but smaller in magnitude compared with the analyses in SHIP. In the fully adjusted model, a SD unit increase in LDL-C, HDL-C, and TG was associated with a −0.001 (95% CI, −0.005 to 0.003; *P* = 0.57), −0.04 (95% CI, −0.044 to −0.036; *P* = 1.88 × 10^−112^), and −0.01 (95% CI, −0.014 to −0.006; *P* = 5.05 × 10^−20^) SD unit change in IGF-I levels, respectively. For the adjusted analyses, the association between HDL-C and TG with IGF-I is directionally consistent but smaller in magnitude compared with the adjusted analyses in SHIP.

### Part IIIB: Two-sample MR analysis to estimate the bi-directional causal association between lipids and IGF-I

We estimated the causal effect of LDL-C, HDL-C, and TG on IGF-I levels using two-sample MR. There was weak evidence that LDL-C or HDL-C affect levels of IGF-I (Table 3). The univariable IVW analyses suggested that a SD unit increase in TG (approximately 81.8 mg/dL) is associated with a −0.13 (95% CI, −0.23 to −0.04; *P* = 0.01) SD unit change in IGF-I. Estimates from sensitivity analyses using methods that take into account potential pleiotropy were in the same direction but differed in magnitude to univariable IVW estimates. Estimates from multivariable IVW (−0.12; 95% CI, −0.20 to −0.05; *P* = 0.002) and MVMR-Egger methods (−0.17; 95% CI, −0.26 to −0.08; *P* = 0.0003) were consistent with the univariable IVW estimates, with weak evidence of directional pleiotropy (intercept = 0.003; SE = 0.001; *P* = 0.054; Table 3). The MR Steiger directionality test suggested that the observed association was directionally causal (Supplementary Table S6).

**Table 3.** β Estimates of SD unit change in IGF-I per SD unit increase in HDL-C, LDL-C, or TG based on two-sample and MVMR analyses.

		Main analysis		Sensitivity analyses									
		Univariable IVW		Univariable weighted median		Univariable MR Egger regression			Multivariable MR		Multivariable Egger		
Exposure	Outcome	*β* ^a^ (95% CI)	*P*	*β* ^a^ (95% CI)	*P*	*β* ^a^ (95% CI)	*P*	Univariable MR Egger intercept (SE; *P*)	*β* ^a^ (95% CI)	*P*	*β* ^a^ (95% CI)	*P*	Multivariable MR Egger intercept (SE; *P*)
LDL-C	IGF-I	0.01 (−0.04 to 0.06)	0.64	0.02 (−0.001 to 0.03)	0.06	−0.01 (−0.09 to 0.06)	0.73	0.002 (0.002; *P* = 0.41)	0.03 (−0.02 to 0.08)	0.28	−0.02 (−0.08 to 0.05)	0.58	0.003 (0.001; *P* = 0.02)
HDL-C	IGF-I	0.02 (−0.04 to 0.08)	0.52	0.004 (−0.02 to 0.02)	0.72	0.04 (−0.05 to 0.14)	0.38	−0.001 (0.002; *P* = 0.53)	−0.02 (−0.08 to 0.05)	0.62	−0.03 (−0.10 to 0.05)	0.48	0.001 (0.001; *P* = 0.59)
TG	IGF-I	−0.13 (−0.23 to −0.04)	0.01	−0.03 (−0.05 to −0.001)	0.04	−0.24 (−0.39 to −0.08)	0.004	0.01 (0.004; *P* = 0.10)	−0.12 (−0.20 to −0.05)	0.002	−0.17 (−0.26 to −0.08)	0.0003	0.003 (0.001, *P* = 0.054)

Abbreviation: *N*, sample size; β, regression coefficient interval.

^a^β refers to the SD unit change in IGF-I levels per 1 SD unit increase in HDL-C, LDL-C, or TG.

In the reverse direction (estimation of the causal effect of IGF-I on lipids), there was little evidence to suggest that IGF-I levels impact circulating LDL-C or HDL-C (Table 4). A SD unit increase in IGF-I (approximately 49.76 ng/mL) was associated with a −0.11 (95% CI, −0.18 to −0.05; *P* = 0.001) SD unit change in TG, using the univariable IVW method. This estimate was larger in magnitude in the univariable MR-Egger analyses (−0.28; 95% CI, −0.42 to −0.15; *P* = 0.00004), with evidence of directional pleiotropy (intercept = 0.01; SE = 0.002; *P* = 0.004). The estimate attenuated towards the null in the weighted median analyses (−0.02; 95% CI, −0.07 to 0.02; *P* = 0.34). The MR Steiger directionality test suggested that the observed association was directionally causal (Supplementary Table S6).

**Table 4.** β^a^ Estimates of SD unit change in LDL-C, HDL-C, and TGs per SD unit increase in IGF-I based on two-sample and MVMR analyses.

		Main Analysis		Sensitivity Analyses				
		IVW		Weighted median		MR Egger regression		MR Egger
Exposure	Outcome	*β* ^a^ (95% CI)	*P*	*β* ^a^ (95% CI)	*P*	*β* ^a^ (95% CI)	*P*	intercept (SE; *P* value)
IGF-I	LDL-C	−0.06 (−0.14 to 0.01)	0.09	−0.02 (−0.08 to 0.03)	0.40	−0.06 (−0.21 to 0.08)	0.39	−0.00002 (0.002; *P* = 0.99)
IGF-I	HDL-C	0.01 (−0.03 to 0.06)	0.59	−0.02 (−0.06 to 0.03)	0.48	0.01 (−0.08 to 0.09)	0.90	0.0002 (0.001; *P* = 0.86)
IGF-I	TG	−0.11 (−0.18 to −0.05)	0.001	−0.02 (−0.07 to 0.02)	0.34	−0.28 (−0.42 to −0.15)	0.00004	0.01 (0.002; *P* = 0.004)

Abbreviation: *N*, sample size.

^a^β refers to the SD unit change in HDL-C, LDL-C, TG, or TC per SD unit change in IGF-I levels.

Supplementary Table S7 shows the estimated genetic correlations between IGF-I with lipid traits. There was little evidence of genetic correlation between IGF-I with the circulating lipids except for HDL-C although the genetic correlation was low [genetic correlation (rG): 0.04; SE = 0.02; *P* = 0.05].

### Part IV: MVMR analyses to estimate the direct effect of lipids/IGF-I on breast cancer

Multivariable IVW analyses were conducted to investigate whether the effect of lipids on overall breast cancer was attenuated following adjustment for IGF-I, or vice versa (Table 5). Using data from BCAC, the MVMR OR for overall breast cancer per SD increase in TG, conditioned on HDL-C, LDL-C, and IGF-I, was 0.95 (95% CI, 0.92–0.99; *P* = 0.03; Table 5), which was comparable with the IGF-I unadjusted model (OR = 0.95; 95% CI, 0.91–0.99; *P* = 0.01; Fig. 2). The MVMR OR for overall breast cancer per SD increase in IGF-I, conditioned on LDL-C, HDL-C, and TG, was 1.09 (95% CI, 1.05–1.14; *P* = 0.0001; Table 5), which was comparable to the lipid unadjusted model (OR = 1.09; 95% CI, 1.04–1.15; *P* = 0.001; Table 2). We assessed likely instrument strength in the MVMR models and found that the conditional *F*-statistics for LDL-C, HDL-C, and TG and IGF-I were 52.87, 40.83, 36.48, and 45.14, respectively, suggesting sufficient instrument strength (Table 5). Evaluation of horizontal pleiotropy using a modified form of Cochran *Q*-statistic with respect to the differences in MVMR estimates across the instruments found evidence of potential pleiotropy in the MVMR model (*P* < 4.31 × 10^−135^).

**Table 5.** Multivariable MR analysis of the direct effect of lipids/IGF-I on breast cancer.

		MVMR			
Exposure	Outcome	*β* (95% CI)	*P*	Conditional *F-*statistic for MVMR	*Q-*statistic for heterogeneity
LDL-C^a^	Overall breast cancer	1.04 (1.00–1.08)	0.05	52.87	*Q* = 2221.84
					*P* = 4.31 × 10^−135^
HDL-C^b^	Overall breast cancer	1.07 (1.03–1.11)	0.0005	40.83	
TG^c^	Overall breast cancer	0.95 (0.92–0.99)	0.03	36.48	
IGF-I^d^	Overall breast cancer	1.09 (1.05–1.14)	0.0001	45.14	

^a^Associations are per 1 SD unit increase in LDL-C when conditioned on IGF-I, HDL-C, and TG.

^b^Associations are per 1 SD unit increase in HDL-C when conditioned on IGF-I, LDL-C, and TG.

^c^Associations are per 1 SD unit increase in TG when conditioned on IGF-I, HDL-C, and LDL-C.

^d^Associations are per 1 SD unit increase in IGF-I when conditioned on TG, HDL-C, and LDL-C.

## Discussion

In this study, we explored the interplay between circulating lipids and IGF-I and the relevance to breast cancer risk. Using two-sample MR, there was strong evidence that HDL-C is positively associated with breast cancer risk, whereas TG is negatively associated with breast cancer risk. Results from observational and MR analyses highlight that TG decreases IGF-I and that IGF-I decreases TG, providing evidence of a bi-directional relationship between TG and IGF-I. The LD score regression analysis contributed towards evidence of a causal relationship between TG and IGF-I, as opposed to shared heritability. Findings from our observational and MR analyses point to an interplay between TG and IGF-I; however, MVMR estimates suggest effects of TG and IGF-I on breast cancer are independent.

The effects of lipids on breast cancer risk have been investigated by several MR studies (8–11), which found consistent evidence in support of HDL-C playing a causal role in the etiology of breast cancer. Conversely, evidence in support of the causal role of LDL-C and TG on breast cancer risk has been less consistent. In agreement with two previous MR studies (8, 11), our study provides evidence that HDL-C is positively associated with overall and ER-positive breast cancer whereas TG is negatively associated with overall breast cancer and ER-positive breast cancer. Findings from our study build on prior investigations through the inclusion of a large female-specific GWAS of lipid traits in UKBB, thus expanding the number of robust female-specific genetic instruments for each lipid trait. As the breast cancer outcome was defined in females, we chose to use female-specific instruments for the lipid traits as sex-specific effects have been observed for these exposures (49). This is in contrast to previous lipid–breast cancer MR studies (8–11), which had used non-sex specific lipid instruments, resulting in reduced precision in their estimates in comparison to our results.

Given the high degree of inter-relatedness among the lipid traits, genetic variants associated with one lipid trait will also be associated with other lipid traits. The main challenge in investigating the association between lipids and breast cancer is addressing the potential for horizontal pleiotropy (violation of the exclusion restriction assumption), which can confound the MR estimates (50). With this in mind, we applied MVMR to simultaneously estimate the direct causal effect of various correlated lipid traits on breast cancer when conditioned on one another in a single model. A MR study on lipid and breast cancer risk using MVMR was recently published (10); the authors included potential confounders such as body mass index and age of menarche in their MVMR model and their primary findings was that both HDL-C and LDL-C were associated with increased breast cancer risk. We did not adjust for potential confounders in our MVMR model to maintain instrument strength (as indicated by the conditional *F*-statistic); however, we conducted MVMR-Egger analyses, which can provide reliable evidence regarding causation even in the presence confounding through unbalanced horizontal pleiotropy. Indeed, results from our MVMR-Egger analyses were consistent with our multivariable IVW analyses.

We investigated the relationship between IGF-I and breast cancer using female-specific instruments from a meta-analysis of two major IGF-I GWAS from UKBB and CHARGE. Our study found evidence that IGF-I increases overall breast cancer, likely driven by ER-positive breast cancer, which is concordant with results from previous observational and MR studies (15, 51). Given that SNPs associated with IGF-I are also associated with other components of the IGF axis, there is a possibility that our results could be biased due to pleiotropy with other components of the IGF axis (52). GWAS analyses for other components of the IGF axis have been conducted (30, 53). However, due to a lack of robust genetic instruments for these individual IGF traits, we were unable to use MVMR to investigate the direct effect of IGF-I conditioned on the other components of the IGF axis. Preclinical evidence suggests that IGF signaling is mitogenic for both ER-positive and ER-negative breast cancer (54). In contrast, our MR results suggest that circulating IGF-I is more influential in ER-positive breast cancer compared with ER-negative breast cancer. However, our MR analyses for ER-negative breast cancer could lack statistical power due to the smaller sample size compared with ER-positive breast cancer. As we have used genetic variants that predict circulating levels of IGF-I, we cannot rule out important aspects of tissue-specific regulation, which may contribute to breast cancer.

Our finding that IGF-I associates with decreased TG is consistent with findings from previous observational (24), interventional (17, 55–57), and candidate gene studies (58, 59). IGF-I can inhibit growth hormone secretion by negative feedback in the growth hormone (GH)–IGF axis. It has been suggested that the effect of circulating IGF-I on circulating TG levels is most likely due its effect on growth hormone or insulin secretion (56, 58, 60). To the best of our best knowledge, this is the first study suggesting that TG levels causally reduce IGF-I levels. The mechanisms by which circulating TGs affect circulating IGF-I levels remain to be elucidated. The liver is the main source of circulating IGF-I (accounting for ∼75% of circulating IGF-I) and many variables are known to control IGF-I synthesis and secretion, including nutrient intake, insulin, and growth hormone levels (61, 62). It is possible that TGs could affect the hepatic synthesis of IGF-I.

Our observational and MR estimates support a bi-directional relationship between TG and IGF-I and suggest that the pathways from these phenotypes to breast cancer overlap. In the context of this, we applied MVMR approaches to investigate the direct effects of TG and IGF-I on breast cancer independently of each other. The associations between TG or IGF-I with breast cancer from the MVMR analyses (wherein LDL-C, HDL-C, TG, and IGF-I were included in the model) was not attenuated when compared with the univariable MR associations. This lack of attenuation is not likely due to weak instrument bias, which is a common problem in MVMR; however, we cannot rule out directional pleiotropy as a source of bias as a modified form of Cochran *Q*-statistic found evidence of potential horizontal pleiotropy in the MVMR model. To this end, further work is required to elucidate other potential modifiable risk factors that drive the putative causal relationship between lipids or IGF-I with breast cancer.

Our study has several limitations. First, despite the large sample size of the UKBB, this cohort is not representative of the general population due to the recruitment of generally healthier individuals with higher socioeconomic status. Hence, our findings might be prone to selection bias and cannot be generalized to the UK population. Second, our study was focused primarily on individuals of European ancestry. Although population homogeneity eliminates population admixture as a potential confounder in our analyses, the findings drawn from this study might not be generalizable to non-European populations.

### Conclusion

In conclusion, our findings highlight a causal role for HDL-C, TG, and IGF-I in breast cancer risk. Observational and bi-directional MR analyses support an interplay between IGF-I and TG; however, results from MVMR analyses suggest that TG and IGF-I may act independently to influence breast cancer. These relationships should be considered in the development of prevention strategies for breast cancer, where interventions are known to modify circulating lipid and IGF traits

## Authors' Disclosures

T.G. Richardson reports employment with Novo Nordisk outside of this work. L.J. Corbin reports grants from Wellcome Trust during the conduct of the study. J.I. Rotter reports grants from NIH during the conduct of the study. No disclosures were reported by the other authors.

## Data Availability

Results of the female-specific lipid GWAS and the IGF-I GWAS will be made available to download from the OpenGWAS database (http://gwas.mrcieu.ac.uk).
