# Supplementary material for: Investigation of the interplay between circulating lipids and IGF-I and relevance to breast cancer risk: an observational and Mendelian randomization study
Source: Cancer Epidemiol Biomarkers Prev. Author manuscript; Available in PMC 2021 Dec 5. (PMC7612074; doi:10.1158/1055-9965.EPI-21-0315)
Supplement: Supplementary file 1 [file EMS136027-supplement-Supplementary_Methods.docx]

**Supplementary Methods** 

**Study populations and data sources**

Study of Health in Pomerania (SHIP)

The Study of Health in Pomerania (SHIP) is a cross-sectional survey in West Pomerania, the north-eastern area of Germany^1^. A sample from the population aged 20 to 79 years was drawn from population registries. First, the three cities of the region (with 17,076 to 65,977 inhabitants) and the 12 towns (with 1516 to 3044 inhabitants) were selected, and then 17 out of 97 smaller towns (with less than 1500 inhabitants), were drawn at random. Second, from each of the selected communities, subjects were drawn at random, proportional to the population size of each community and stratified by age and gender. Only individuals with German citizenship and main residency in the study area were included. 7008 subjects aged 20 to 79 years were sampled, with 292 persons of each gender in each of twelve five-year age strata.  The net sample (without out-migrated or deceased persons) comprised 6267 eligible subjects, of whom 4308 eventually participated in the baseline SHIP study (SHIP-0) with a response rate of 68.8%. Of these subjects, individuals with missing genotype or phenotype data were excluded.  Valid data were available in 3812 individuals of the SHIP-0 population. At the SHIP-0 visit (baseline), non-fasting serum IGF-I concentrations were determined by automated two-site chemiluminescence immunoassays (Nichols Advantage®; Nichols Institute Diagnostica GmbH, Bad Vilbel, Germany).  In both assays, in the first step biotin labelled monoclonal antibodies were employed as capture, and in the second step the acridinium-ester labelled monoclonal antibodies were used for detection.  To determine IGF-I concentration the samples were acidified to separate IGF-I from IGF-binding proteins.  The analytical sensitivity was 6 ng/mL.  The IGF-I assay has been calibrated against the World Health Organization International Reference Reagent 1988, Insulin-like Growth Factor-I 87/518. Total cholesterol and HDL concentrations were measured photometrically (Hitachi 704, Roche, Mannheim, Germany), and serum LDL was measured by applying a precipitation procedure using dextran sulphate (Immuno, Heidelberg, Germany) on an Epos 5060 (Eppendorf, Hamburg, Germany). The serum aliquots were prepared for immediate analysis and for storage at -80 °C in the Integrated Research Biobank (Liconic, Liechtenstein).

IGF working group of the CHARGE consortium 

A recent GWAS meta-analysis for IGF-I (n=30,884 individuals of European descent) conducted by the IGF working group of the CHARGE consortium identified 7 SNPs that reached genome-wide significance (p<5x10^-8^) for circulating IGF-I levels^2^. The per-allele effect of each SNP on IGF-I was reported (in SD units). An SD unit of IGF-I is equivalent to 57.6 ng/ml (calculated using data from the SHIP cohort). The methods applied to measure IGF-I levels differed among the participating cohorts and include enzyme-linked immunosorbent assays (ELISA), radioimmunoassays (RIA) and chemiluminescent immunoassays, as previously described^2^. Participants were genotyped on genome-wide arrays and SNPs were imputed using the HapMap2 reference panel. Association analyses in individual studies were performed on IGF-I levels measured in ng/mL using a multiple linear regression with an additive genetic model based on allele dosages adjusted for age and stratified by sex. All cohorts accounted for relatedness, population substructure using genetic principal components, study centre and laboratory batch of IGF-I measurement where applicable. Individuals of non-European ancestry, with missing phenotype data, a diagnosis of growth hormone deficiency or known use of human growth hormones were excluded from the analyses. Betas and SEs were estimated from the p-values using a previously described method^3^. As an approximation, the phenotypic SD was set to 1, resulting in an effect change per unit SD IGF-I.

 

Global lipids consortium (GLGC)

The most recent GWAS meta-analysis conducted by the Global Lipids Genetic Consortium (GLCL) and Do et al. identified 185 independent SNPs (linkage disequilibrium r^2^<0.05) (in 157 loci) that met genome-wide significance (p<5x10^-8^) for circulating HDL-C, LDL-C and TG in a sample of 188,577 individuals of European descent^4,5^. These SNPs explained approximately 13.7%, 14.6% and 11.7% of the variance in circulating levels of HLD-C, LDL-C and TG, respectively. Blood lipid levels were typically measured after >8hr of fasting. Individuals taking lipid-lowering medications were excluded when possible. In most studies, total cholesterol, HDL-C and TG were measured after >8hr of fasting. LDL cholesterol levels were directly measured in 10 studies and were estimated using the Friedewald formula in the remaining studies^6^. The per-allele effect of each SNP on each lipid trait was reported in SD units based on inverse normal transformed residuals of lipid concentrations, after adjustment for age and sex. An SD unit of LDL-C, HDL-C and TG on the observed scale is equivalent to approximately 39.9mg/dL, 17.9mg/dL and 81.8mg/dL, respectively^7^. Participants were genotyped using the Illumina iSelect Metabochip genotyping array. Individual SNP association tests were performed using linear regression with the inverse normal transformed trait values as the dependent variable and the expected allele count for each individual as the independent variable. Meta-analysis was performed using the Stouffer method with weights proportional to the square root of the sample size for each sample. 

Breast Cancer Association Consortium (BCAC)

The BCAC consortium conducted a GWAS meta-analysis to identify SNPs associated with overall (133,384 breast cancer (BCa) cases and 113,789 controls), ER-positive (69,501 BCa cases and 105,974 controls) and ER-negative (21,468 BCa cases and 105,974 controls) BCa risk^8,9^. Genotyping of BCAC samples were performed using an Illumina Custom Infinium genotyping array (OncoArray) consisting of ~570 000 single nucleotide polymorphisms (SNPs) and imputed using the 1000 Genomes Project as a reference panel as previously described^10^. All SNPs with poor imputation quality (r^2^<0.30), a minor allele frequency of <1%, a call rate of <95% or evidence of Hardy-Weinberg equilibrium were removed. All participating studies were approved by their appropriate ethics review board and all individuals provided informed consent. 

UK Biobank

UK Biobank is a population-based health research resource consisting of approximately 500 000 people, aged between 38 years and 73 years, who were recruited between the years 2006 and 2010 from across the UK^11^. Particularly focused on identifying determinants of human diseases in middle- aged and older individuals, participants provided a range of information (such as demographics, health status, lifestyle measures, cognitive testing, personality self-report, and physical and mental health measures) via questionnaires and interviews; anthropometric measures, BP readings and samples of blood, urine and saliva were also taken (data available at www.ukbiobank.ac.uk). A full description of the study design, participants and quality control (QC) methods have been described in detail previously^12^. The full data release contains the cohort of successfully genotyped samples (n=488,377). 49,979 individuals were genotyped using the UK BiLEVE array and 438 398 using the UK Biobank axiom array. Pre-imputation QC, phasing and imputation are described elsewhere^13^. In brief, prior to phasing, multiallelic SNPs or those with MAF ≤1% were removed. Phasing of genotype data was performed using a modified version of the SHAPEIT2 algorithm^14^. Genotype imputation to a reference set combining the UK10K haplotype and HRC reference panels^15^ was performed using IMPUTE2 algorithms^16^. The analyses presented here were restricted to autosomal variants within the HRC site list using a graded filtering with varying imputation quality for different allele frequency ranges^17^. Therefore, rarer genetic variants are required to have a higher imputation INFO score (Info>0.3 for MAF >3%; Info>0.6 for MAF 1-3%; Info>0.8 for MAF 0.5-1%; Info>0.9 for MAF 0.1-0.5%) with MAF and Info scores having been recalculated on an in-house derived ‘European’ subset^17^. Individuals with sex-mismatch (derived by comparing genetic sex and reported sex) or individuals with sex-chromosome aneuploidy were excluded from the analysis (n=814). We restricted the sample to individuals of ‘European’ ancestry as defined by an in-house k-means cluster analysis performed using the first 4 principal components provided by UK Biobank in the statistical software environment R. The current analysis includes the largest cluster from this analysis (n=462,933)^17^. 

**References**

1. 	Volzke H, Alte D, Schmidt CO, et al. Cohort Profile: The Study of Health in Pomerania. *Int J Epidemiol*. 2011 Apr;**40**(2):294–307. 

2. 	Teumer A, Qi Q, Nethander M, et al. Genomewide meta-analysis identifies loci associated with IGF-I and IGFBP-3 levels with impact on age-related traits. *Aging Cell*. 2016 Oct;**15**(5):811–824. 

3. 	Rietveld CA, Medland SE, Derringer J, et al. GWAS of 126,559 Individuals Identifies Genetic Variants Associated with Educational Attainment. *Science (80- )*. 2013 Jun;**340**(6139):1467–1471. 

4. 	Do R, Willer CJ, Schmidt EM, et al. Common variants associated with plasma triglycerides and risk for coronary artery disease. *Nat Genet*. 2013;**45**(11):1345–1352. 

5. 	Willer CJ, Schmidt EM, Sengupta S, et al. Discovery and refinement of loci associated with lipid levels. *Nat Genet*. 2013 Nov;**45**(11):1274–1283. 

6. 	Friedewald WT, Levy RI, Fredrickson DS. Estimation of the concentration of low-density lipoprotein cholesterol in plasma, without use of the preparative ultracentrifuge. *Clin Chem*. 1972/06/01. 1972;**18**(6):499–502. 

7. 	Willer CJ, Schmidt EM, Sengupta S, et al. Discovery and refinement of loci associated with lipid levels. *Nat Genet*. 2013;**45**(11):1274–1283. 

8. 	Michailidou K, Lindström S, Dennis J, et al. Association analysis identifies 65 new breast cancer risk loci. *Nature*. 2017; 

9. 	Zhang H, Ahearn TU, Lecarpentier J, et al. Genome-wide association study identifies 32 novel breast cancer susceptibility loci from overall and subtype-specific analyses. *Nat Genet*. 2020; 

10. 	Amos CI, Dennis J, Wang Z, et al. The OncoArray Consortium: a Network for Understanding the Genetic Architecture of Common Cancers. *Cancer Epidemiol Prev Biomarkers*. 2016;**26**(1):126–135. 

11. 	Allen NE, Sudlow C, Peakman T, Collins R, UK Biobank. UK Biobank Data: Come and Get It. *Sci Transl Med*. 2014 Feb;**6**(224):224ed4-224ed4. 

12. 	Collins R. What makes UK Biobank special? *Lancet*. 2012 Mar;**379**(9822):1173–1174. 

13. 	Bycroft C, Freeman C, Petkova D, et al. Genome-wide genetic data on ~500,000 UK Biobank participants. *bioRxiv*. 2017 Jul; 

14. 	O’Connell J, Sharp K, Shrine N, et al. Haplotype estimation for biobank-scale data sets. *Nat Genet*. Nature Publishing Group; 2016 Jul;**48**(7):817–820. 

15. 	Huang J, Howie B, McCarthy S, et al. Improved imputation of low-frequency and rare variants using the UK10K haplotype reference panel. *Nat Commun*. Nature Publishing Group; 2015 Nov;**6**(1):8111. 

16. 	Howie B, Marchini J, Stephens M. Genotype Imputation with Thousands of Genomes. *G3&amp;#58; Genes|Genomes|Genetics*. 2011 Nov;**1**(6):457–470. 

17. 	Mitchell, R, Hemani, G, Dudding, T, Paternoster L. UK Biobank Genetic Data: MRC-IEU Quality Control, Version 1. 2017; 

a) 

**ER positive**

**OR (95%CI)**

b) 

**ER negative**

**OR (95%CI)**

TG

LDL-C

HDL-C

0.94 (0.89, 0.99)

1.02 (0.96, 1.07)

0.99 (0.91, 1.07)

1.02 (0.96, 1.09)

)

1.04 (0.99, 1.08)

1.02 (0.97, 1.07)

1.07 (1.01, 1.12)

1.10 (1.02, 1.19)

1.06 (0.99, 1.13)

)

1.06 (1.01, 1.10)

1.08 (1.03, 1.14)

0.87 (0.79, 0.95)

0.91 (0.85, 0.97)

)

0.94 (0.90, 0.99)

0.90 (0.85, 0.95)

0.94 (0.87, 1.01)

0.97 (0.91, 1.05)

0.94 (0.85, 1.04)

0.96 (0.87, 1.06)

)

1.00 (0.95, 1.05)

0.98 (0.93, 1.05)

1.10 (1.04, 1.17)

1.08 (0.99, 1.19)

1.05 (0.96, 1.15)

)

1.08 (1.03, 1.14)

1.08 (1.01, 1.15)

0.89 (0.79, 1.00)

0.90 (0.81, 0.99)

)

0.97 (0.92, 1.03)

0.96 (0.89, 1.04)

**Supplementary Figure 1.** Estimates of the causal relationship between circulating lipid traits and breast cancer risk stratified by ER status. 

The forest plot shows the estimate of the causal effect of LDL-C, HDL-C and TG on a) ER positive (n=69,501 cases and 105,974 controls) and b) ER negative (n=21,468 cases and 105,974 controls) breast cancer risk using summary data from the breast cancer association consortium (BCAC) Circles represent point estimates from individual analyses. Horizontal lines represent the 95% confidence intervals. 

Supplementary Table 1. Genetic instruments for lipid traits used in multivariable MR analyses – see excel file

## Supplementary Table 2. Genetic instruments for IGF-I – see excel file

**Supplementary Table 3.** Study characteristics of the SHIP and UKBB cohort included in cross-sectional observational analyses 

|  | **SHIP** **(N=3812)** | **UK Biobank** **(N=438,988)** |
| --- | --- | --- |
| **Trait** | **Mean (SD) / n (%)** |  |
| **Age (yrs)** | 49.83 (16.26) | 56.55 (8.09) |
| **Sex (male)** | 1866 (48.95%) | 195,222 (46.22%) |
| **Current smoking** | 1147 (30.09%) | 44,578 (23.39%) |
| **Diabetes** | 416 (10.91%) | 21,995 (5.2%) |
| **BMI (kg/m**^**2**^**)** | 27.3 (4.78) | 27.43 (4.78) |
| **LDL-C (mmol/l)** | 3.57 (1.16) | 3.56 (0.87) |
| **HDL-C (mmol/l)** | 1.45 (0.44) | 1.45 (0.38) |
| **TG (mmol/l)** | 1.82 (1.30) | 1.75 (1.03) |

BMI, body mass index; yrs, years; kg, kg/m2, kilograms per metres squared; LDL-C, low density lipoprotein; 

HDL-C, high density lipoprotein; TG, triglycerides;SD, standard deviation 

**Supplementary Table 4.** Beta estimates of SD unit change in IGF-I per SD unit increase in HDL-C, LDL-C or TG based on observational analyses in SHIP and UK Biobank.

|  |  |  | **SHIP** |  |  |  | **UK Biobank** |  |  |  |  |
| --- | --- | --- | --- | --- | --- | --- | --- | --- | --- | --- | --- |
|  |  |  | **Unadjusted** |  | **Adjusted**^**a**^ |  |  | **Unadjusted** |  | **Adjusted**^**a**^ |  |
| **Exposure** | **Outcome** | **N** | **Beta**^**b**^ **(95%CI)** | ***p*** | **Beta**^**b**^ **(95%CI)** | ***p*** | **N** | **Beta**^**b**^ **(95%CI)** | ***p*** | **Beta**^**b**^ **(95%CI)** | ***p*** |
| **LDL-C** | **IGF-I** | 3812 | -0.11 (-0.14, -0.08) | 2x10^-12^ | 0.03 (0.004, 0.06) | 0.03 | 422,389 | -0.01 (-0.014, -0.006) | 2.08x10^-19^ | -0.001 (-0.005, 0.003) | 0.57 |
| **HDL-C** | **IGF-I** | 3812 | 0.02 (-0.01, 0.05) | 0.29 | -0.05 (-0.08, -0.02) | 0.001 | 422,389 | -0.03 (-0.032, -0.028) | 8.9x10^-102^ | -0.04 (-0.044, -0.036) | 1.88x10^-112^ |
| **TG** | **IGF-I** | 3812 | -0.16 (-0.19, -0.13) | 2x10^-22^ | -0.06 (-0.09, -0.04) | 1.5x10^-5^ | 422,389 | -0.05 (-0.054, -0.046) | 9.37x10^-204^ | -0.01 (-0.014, -0.006) | 5.05x10^-20^ |

N, sample size; Beta, regression coefficient; 95% CI, 95% confidence interval; *p*, p-value; HDL-C, high density lipoprotein cholesterol; LDL-C, low density lipoprotein cholesterol; TG, triglycerides; IGF-I, insulin-like growth factor 1

^a^Analyses were adjusted for age, gender, smoking status, body mass index and diabetes status

^**b**^Beta refers to the SD unit change in IGF-I per 1 SD unit increase in HDL-C, LDL-C or TG.

**Supplementary Table 5.** Association between IGF-I or lipid traits and potential confounders measured in the SHIP study.

|  | **IGF-I** |  | **LDL** |  | **HDL** |  | **Triglycerides** |  |
| --- | --- | --- | --- | --- | --- | --- | --- | --- |
|  | **Beta** **(95%CI)** | ***p*** | **Beta** **(95%CI)** | ***p*** | **Beta** **(95%CI)** | ***p*** | **Beta** **(95%CI)** | ***p*** |
| **Age** | -0.03 (-0.03, -0.03) | 2.87x10^-249^ | 0.02 (0.01, 0.02) | 1.79X10^-61^ | -0.005 (-0.007, -0.003) | 2.19x10^-7^ | 0.01 (0.01, 0.01) | 4.17x10^-24^ |
| **Gender** | 0.05 (-0.01, 0.12) | 0.10 | -0.10 (-0.16, -0.04) | 0.002 | 0.67 (0.61, 0.73) | 2.93x10^-100^ | -0.41 (-0.47, -0.34) | 9.5x10^-37^ |
| **BMI** | -0.05 (-0.05, -0.04) | 2.51x10^-42^ | 0.04 (0.03, 0.044) | 5.33x10^-27^ | -0.06 (-0.06, -0.05) | 1.42x10^-65^ | 0.06 (0.05, 0.07) | 7.5x10^-79^ |
| **Smoking status** | 0.28 (0.22, 0.35) | 6.27x10^-16^ | -0.02 (-0.09, 0.05) | 0.05 | -0.10 (-0.17, -0.04) | 0.003 | 0.05 (-0.02, 0.12) | 0.12 |
| **Diabetes status** | -0.53 (-0.63, -0.42) | 1.77x10^-24^ | 0.01 (-0.09, 0.12) | 0.80 | -0.50 (-0.60, -0.40) | 1.98x10^-22^ | 0.60 (0.50, 0.70) | 7.82x10^-31^ |

Beta, effect size; CI, confidence interval; *p*, p-value; IGF-I, insulin-like growth factor 1; LDL-C, low-density lipoprotein; HDL-C, high-density lipoprotein; BMI, body mass index.

^a^Beta refers to unit change in IGF-I (ng/ml), LDL-C (mmol/l), HDL-C (mmol/l) and triglycerides (mmol/l) levels per unit increase in confounders. Calculated using unadjusted linear regression. 

**Supplementary Table 6.** MR Steiger directionality test

| **Exposure** | **Outcome** | **Exposure sample size** | **Outcome sample size** | **SNP R**^**2**^ **for exposure** | **SNP R**^**2**^ **for outcome** | **Correct causal direction** |
| --- | --- | --- | --- | --- | --- | --- |
| LDL-C | IGF-I | 94,311 | 438,988 | 0.12 | 0.005 | True |
| HDL-C | IGF-I | 94,311 | 438,988 | 0.11 | 0.007 | True |
| TG | IGF-I | 94,311 | 438,988 | 0.14 | 0.008 | True |
| IGF-I | LDL-C | 438,988 | 94,311 | 0.08 | 0.01 | True |
| IGF-I | HDL-C | 438,988 | 94,311 | 0.08 | 0.01 | True |
| IGF-I | TG | 438,988 | 94,311 | 0.08 | 0.02 | True |

R^2 ^values are approximate; R^2^, proportion of variance; IGF-I, insulin-like growth factor 1; LDL-C, low-density lipoprotein; HDL-C, high-density lipoprotein; TG, triglycerides; correct causal direction, the inferred causal direction between exposures and outcome

**Supplementary Table 7.** Genetic correlation between IGF-I and lipid traits

| **Trait 1** | **Trait 2** | **Rg** | **se** | ***p*** |
| --- | --- | --- | --- | --- |
| LDL-C | IGF-I | -0.005 | 0.03 | 0.88 |
| HDL-C | IGF-I | 0.04 | 0.02 | 0.05 |
| TG | IGF-I | -0.05 | 0.03 | 0.16 |

IGF-I, insulin-like growth factor 1; LDL-C, low-density lipoprotein; HDL-C, high-density lipoprotein; Rg, genetic correlation; se, standard error; *p*, p-value
